# Controlling sound radiation through an opening with secondary loudspeakers along its boundaries

**DOI:** 10.1038/s41598-017-13546-2

**Published:** 2017-10-17

**Authors:** Shuping Wang, Jiancheng Tao, Xiaojun Qiu

**Affiliations:** 1 Key Laboratory of Modern Acoustics and Institute of Acoustics, Nanjing University, Nanjing, China; 2 School of Electrical, Mechanical, and Mechatronic System, University of Technology Sydney, Sydney, Australia

## Abstract

We propose a virtual sound barrier system that blocks sound transmission through openings without affecting access, light and air circulation. The proposed system applies active control technique to cancel sound transmission with a double layered loudspeaker array at the edge of the opening. Unlike traditional transparent glass windows, recently invented double-glazed ventilation windows and planar active sound barriers or any other metamaterials designed to reduce sound transmission, secondary loudspeakers are put only along the boundaries of the opening, which provides the possibility to make it invisible. Simulation and experimental results demonstrate its feasibility for broadband sound control, especially for low frequency sound which is usually hard to attenuate with existing methods.

## Introduction

Openings like doors, windows and vents of air conditioning systems are commonly used in buildings to keep access, light and ventilation, but unwanted sound transmits via the openings at the same time. Traditional methods like porous materials, microperforated panels have been used to attenuate noise for a long time^1–3^. Recently, rapid development of metamaterials provides another option for noise control by designing novel materials with high sound absorption coefficients^4–19^. Materials with negative modulus or negative density might have applications in this field as well^20–22^. However, these structures or materials have to be implemented over the entire acoustic transmission path to achieve effective sound reduction, so they are not suitable to be used to reduce sound radiation through openings as they result in the block of air and light.

Compared with passive methods mentioned above, active noise control provides an alternative way to reduce sound transmission, especially low frequency sound, when there are volume, weight and/or aesthetic constraints^23,24^. Active Acoustic Shielding (AAS), double-glazed ventilation windows and planar active sound barriers have all been proposed to reduce sound radiation through openings^25–28^. In this work, we propose a virtual sound barrier different from existing active noise control systems. Our system uses a double layered loudspeaker array only at the edge of the opening, which is beneficial to access, light and air circulation through the opening. Previous work has shown that single layered loudspeakers cannot achieve global control of sound radiation through openings at relatively high frequencies^29^, but the double layered loudspeaker system we propose can block sound transmission through openings effectively over a wide frequency band and it is demonstrated by simulations and experiments.

## Results

**Figure 1:**
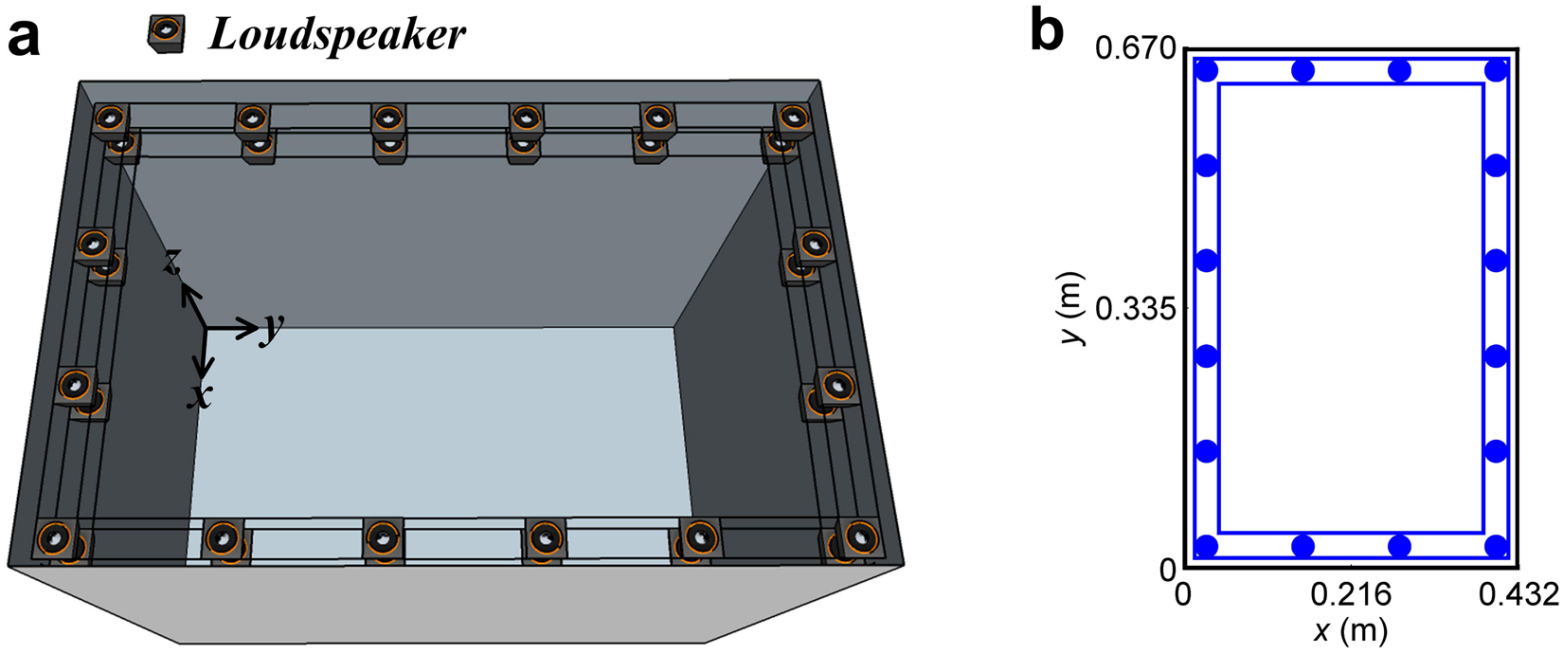
**(a)** Schematic diagram of the double layered secondary loudspeaker system. (**b**) Positions of 16 out of the 32 loudspeakers in *x*-*y* plane in the virtual sound barrier system.

**Figure 2:**
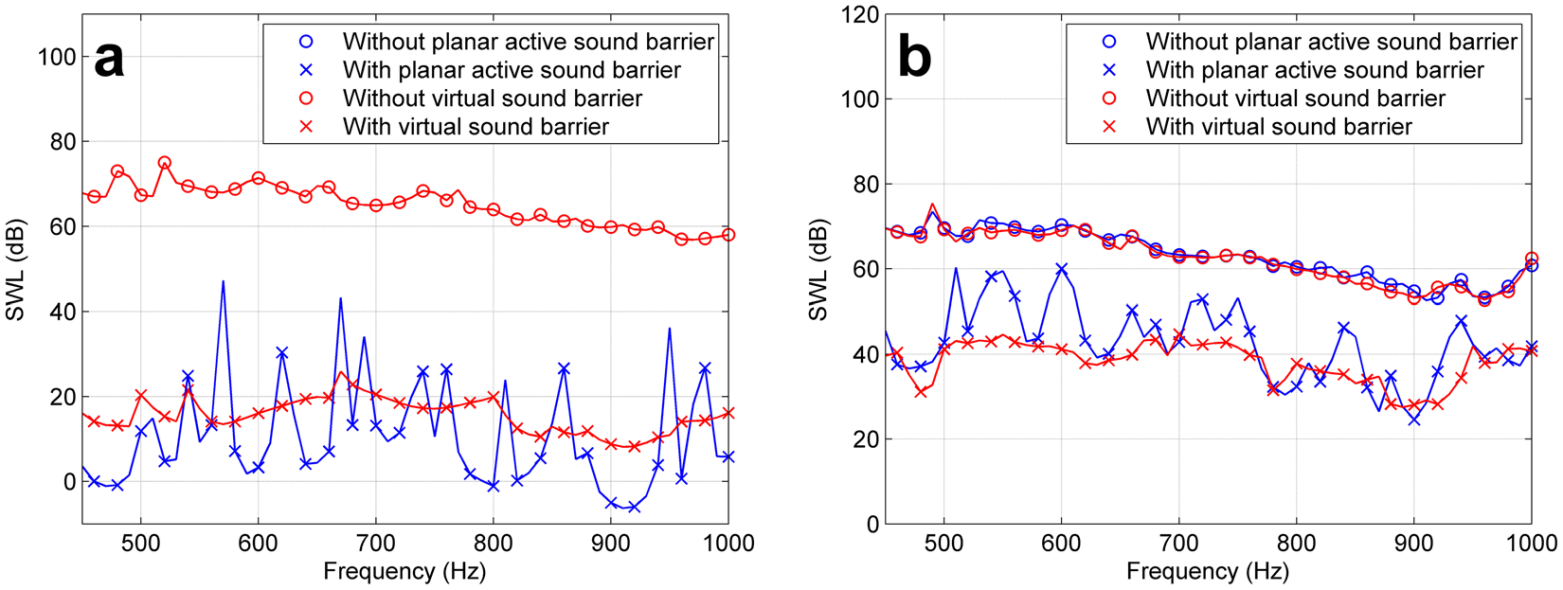
The sound power level (SWL) without and with the proposed virtual sound barrier system and the existing planar active sound barrier system. (**a**) Simulation results. (**b**) Experimental results.

Figure 1a shows a schematic diagram of the double layered secondary loudspeaker system. A double layered loudspeaker array is fixed at the edge of the opening, and the projections of loudspeakers in both layers are the same. A rigid rectangular cavity of 0.432 m × 0.670 m × 0.598 m (Length × Width × Height) is used as the simulation model, and the opening size is 0.432 m × 0.670 m. The origin of the coordinate is one of the vertex at the bottom of the cavity. In the proposed system, 16 out of the 32 loudspeakers are at the height of 0.448 m while the other 16 are at the height of 0.548 m. Their positions in *x*-*y* plane are shown in Fig. 1b. The primary sound source that generates unwanted sound (to be blocked by the proposed system) is assumed to be at (0.1, 0.1, 0.1) m inside the open cavity. The sound power levels (SWL) radiated outward through the opening without and with the virtual sound barrier system are calculated and shown in Fig. 2a.

In Fig. 2a, the sound power reductions from 450 Hz to 1000 Hz are all more than 40 dB with the virtual sound barrier system which is sufficient for an active noise control system. Since it has been demonstrated that applying a planar active sound barrier system consisting of loudspeakers evenly distributed over the entire opening is an effective way to reduce sound power radiation through openings^30^, simulation results of a planar active sound barrier with 32 loudspeakers are also indicated in Fig. 2a for comparison. More details about the planar active sound barrier system can be found in the supplementary information. It can be observed that there exist some frequencies at which the sound cannot be attenuated well by the planar active sound barrier system while the proposed virtual sound barrier does not have the weakness. For example, the sound reduction at 570 Hz is only 20.7 dB with the planar active sound barrier while it is 54.4 dB at the same frequency with the virtual sound barrier. It demonstrates that the proposed system can achieve more stable sound reduction performance than a planar active sound barrier system over a wide frequency band.

**Figure 3:**
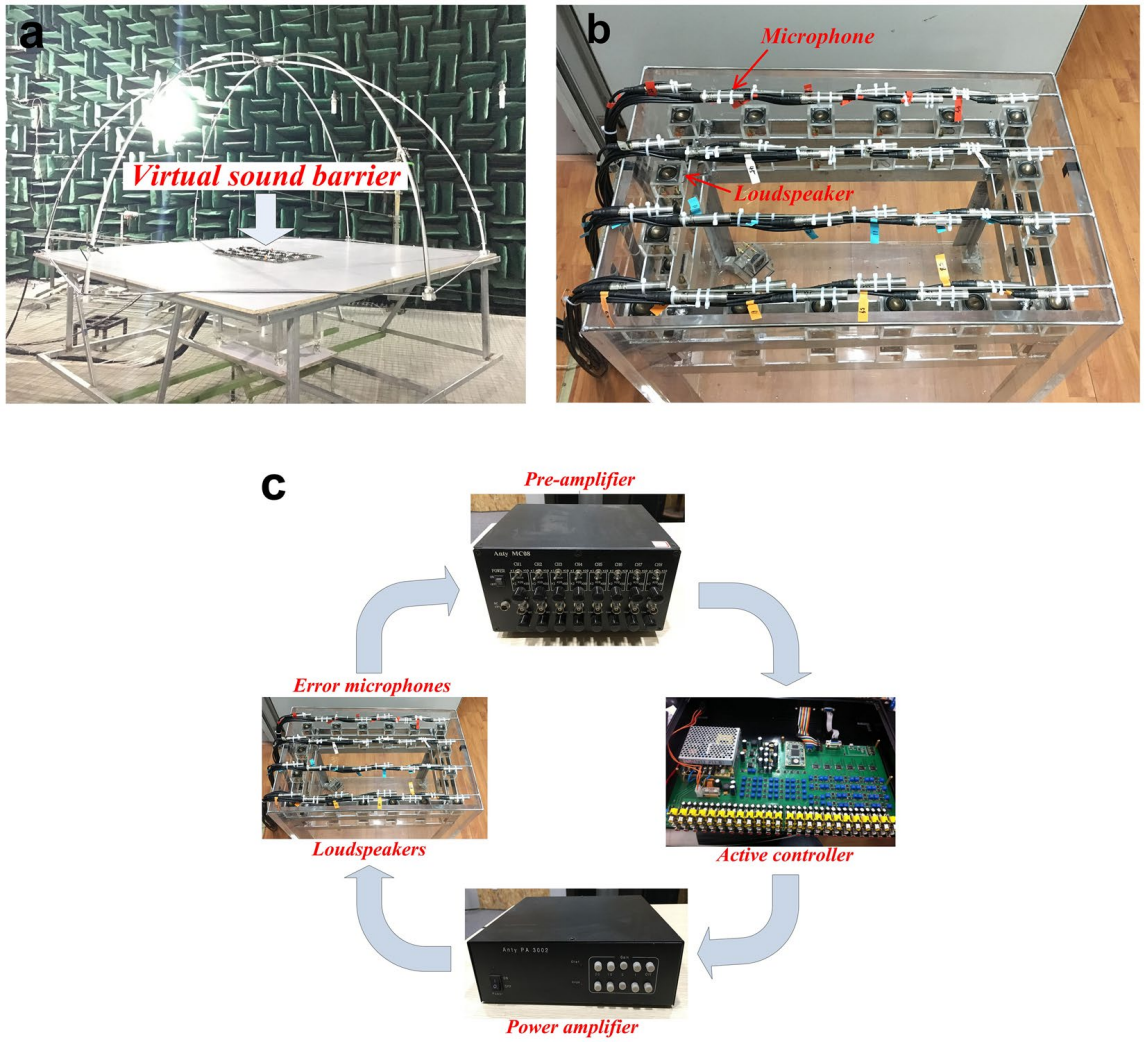
Photos of the experimental setup. (**a**) The panoramic view of the experimental setup in the anechoic room. (**b**) A closer look at the virtual sound barrier system at the opening. (**c**) Schematic diagram of the experimental setup.

The experiments were carried out in the anechoic room of Nanjing University to verify the simulation results. Figure 3 shows the experimental setup. An open cavity of the same size as in the simulations is constructed with five 20 mm-thick acrylic glass plates which provide sufficient sound transmission loss at frequencies of interest, thus sound outside the cavity is solely that transmitted through the opening. 32 error microphones were evenly distributed over the entire opening to pick up the error signals in the experiments, as shown in Fig. 3b, but there are different error sensor strategies to remove the error microphones from the opening to make the virtual sound barrier invisible (see supplementary information for more details). The error signals amplified by the pre-amplifiers were fed to the active controller, which generated outputs to the power amplifiers to drive the loudspeakers after signal processing, as shown in Fig. 3c. The sound power level was measured according to ISO 3744 with a B&K PULSE 3560D multichannel Analyzer^31^. 10 microphones were fixed at the hemisphere frame with a radius of 1.5 m to measure the total sound power level of the system. The primary sound source inside the open cavity generated tonal signal of a certain frequency at a time. The positions of loudspeakers were also the same as adopted in the simulations. The waveform synthesis algorithm was applied by using a commercial active controller^32,33^. It uses a feedforward structure which is an inherently stable system^23^.

Sound power levels were measured without and with the virtual sound barrier system, and the results are shown in Fig. 2b together with the results of the 32-channel planar active sound barrier system. Due to the background noise and dynamic range of the active controller, noise reduction in experiments is much less than that in the simulations, but the trends of the curves in Fig. 2b are similar to the simulation curves in Fig. 2a, and it leads to similar conclusions as in simulations. The planar active sound barrier with loudspeakers evenly distributed over the entire opening achieved good sound reduction performance at some frequencies, but at some other frequencies like 510 Hz, the system is hard to converge which results in much less sound power reduction of only about 5 dB. The proposed virtual sound barrier system achieved a stable sound power reduction of more than 15 dB at most frequencies below 1000 Hz. The system is also effective when the primary sound field is general and more complicated because a complicated sound source can be decomposed into the summation of a number of point sources. We also did the active control experiments when there are more primary sources emitting sound energy at different frequencies simultaneously, and the results show that the system is still effective (see supplementary information).

## Discussion

**Figure 4:**
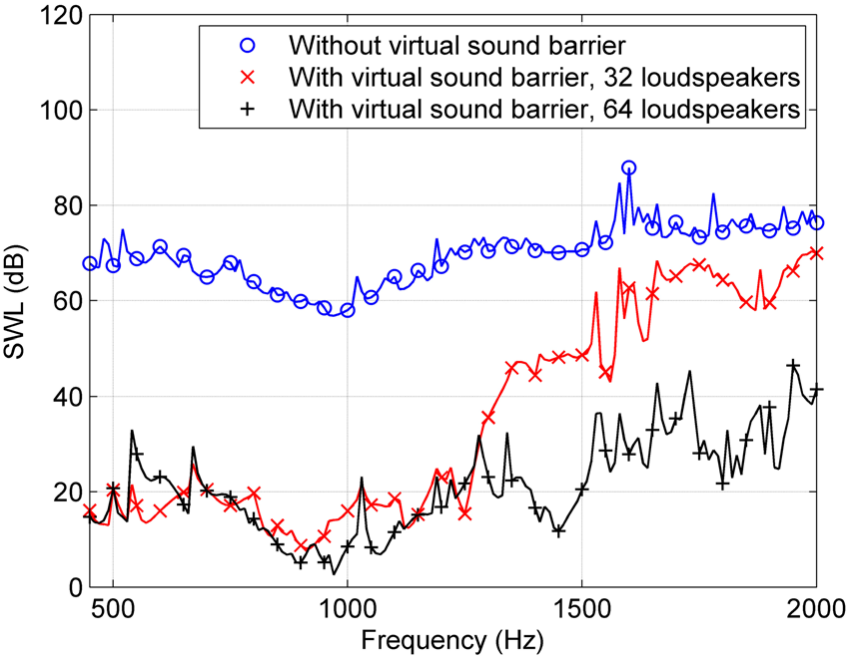
The simulation sound power levels without and with the proposed virtual sound barrier system with different numbers of loudspeakers.

If more loudspeakers are applied in the virtual sound barrier system, sound reduction at higher frequencies can be achieved. Figure 4 shows the simulation sound power levels when there are 32 and 64 loudspeakers at the edge, 16 and 32 loudspeakers at both layers, respectively. The two layers are at the height of 0.448 m and 0.548 m in the 32-channel virtual sound barrier system and 0.498 m and 0.548 m in the 64-channel system. It can be seen from Fig. 4 that while sound power reduction achieved by the 32-channel virtual sound barrier system from 1500 Hz to 2000 Hz is limited, sound in this frequency band can be attenuated by more than 28 dB by the 64-channel virtual sound barrier system. The application of the proposed system can be extended to even higher frequencies with a larger number of loudspeakers, an appropriate distance between two layers or a multi-layered loudspeaker array at the edge of the opening.

**Figure 5:**
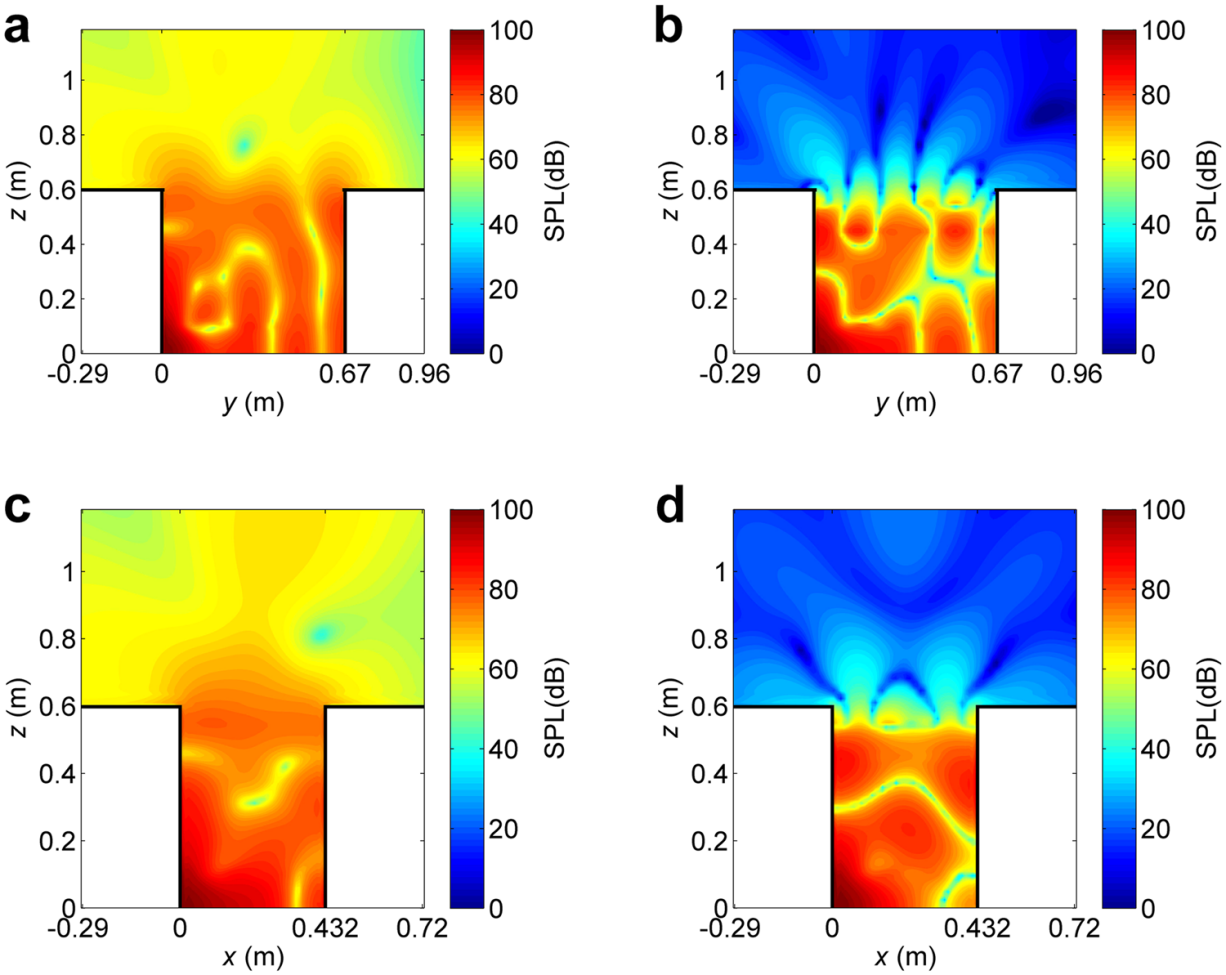
The sound pressure level in *y*-*z* and *x*-*z* plane. (**a**) Without virtual sound barrier, *x* = 0.03 m plane. (**b**) With virtual sound barrier, *x* = 0.03 m plane. (**c**) Without virtual sound barrier, *y* = 0.03 m plane. (**d**) With virtual sound barrier, *y* = 0.03 m plane.

The sound power of the primary sound source and secondary loudspeakers at 1000 Hz without and with the virtual sound barrier are listed in Table 1. The sound power of the primary sound source is significantly reduced (from 2.5 × 10^−4^ W to 6.6 × 10^−8^ W) when the virtual sound barrier is working, which indicates that the main mechanism of active control is unloading the primary source. Figure 5 shows the sound pressure level without and with the virtual sound barrier in a *y*-*z* or *x*-*z* plane in and outside the open cavity. It is clear that the sound pressure level outside the open cavity (proportional to the total sound power of the system) is significantly reduced, while inside the cavity, the sound pressure level remains the same or even increases.

**Table 1:** The sound power of the primary sound source and the total sound power of secondary loudspeakers at 1000 Hz without and with the virtual sound barrier.

Sound power (W)	The primary sound source	Secondary loudspeakers
Without virtual sound barrier	2.5 × 10^−4^	0.0
With virtual sound barrier	6.6 × 10^−8^	−5.0 × 10^−8^

In conclusion, we propose a virtual sound barrier system and demonstrate its feasibility to control sound transmission through openings over a wide frequency band by simulations and experiments. As the secondary sources are at the edge instead of in the pathway of the opening, the proposed system attenuates sound transmission with little effect on ventilation, lighting and access, which is almost impossible to achieve with current passive or active sound control methods. The performance of the proposed system is better than existing active control methods such as planar active sound barriers, because its performance is more stable over a wide frequency range and it overcomes the difficulty of convergence that happens at some frequencies in a planar active sound barrier system. The proposed system is easier to implement and has significant practical application potentials. With a combination of the proposed configuration of secondary loudspeakers and proper error sensor locations, the system can be completely invisible.

## Methods

### Simulations

To evaluate the performance of the proposed virtual sound barrier system, the cost function is defined as the sound power of the system plus a weighted control power^34^

1 $$J=\frac{1}{2}\{\mathrm{Re}[{q}_{{\rm{p}}}^{{\rm{H}}}{p}_{{\rm{p}}}]+\mathrm{Re}[{{\boldsymbol{q}}}_{{\rm{s}}}^{{\rm{H}}}{{\boldsymbol{p}}}_{{\rm{s}}}]\}+\beta {{\boldsymbol{q}}}_{{\rm{s}}}^{{\rm{H}}}{{\boldsymbol{q}}}_{{\rm{s}}},$$

where *q*_p_ is the strength of the primary sound source, ***q***_s_ is the strength vector of the secondary loudspeakers, *p*_p_ is the sound pressure at the position of the primary sound source and ***p***_s_ is the sound pressure vector at the positions of secondary loudspeakers. The transcript H denotes the Hermitian transpose and Re[] means the real part of the value in the square brackets. *β* is a positive real number to constrain the strengths of secondary loudspeakers^28,35^. By minimizing the cost function in Eq. (1), the optimized strengths of secondary loudspeakers can be obtained^34^

2 $${{\boldsymbol{q}}}_{s}=-{({{\boldsymbol{R}}}_{{\rm{s}}{\rm{s}}}+\beta {\bf{I}})}^{-1}{{\boldsymbol{R}}}_{{\rm{s}}{\rm{p}}}{q}_{{\rm{p}}},$$

where ***R***_sp_ = Re[***Z***_sp_], ***R***_ss_ = Re[***Z***_ss_]. ***Z***_sp_ is the acoustic transfer function vector between the primary sound source and secondary loudspeakers, ***Z***_ss_ is the acoustic transfer function matrix between the secondary loudspeakers, and **I** is an identity matrix.

It is assumed that the opening is embedded at an infinite rigid baffle as it is quite complicated to investigate the sound field in and outside an unbaffled opening analytically^36,37^. Since *q*_p_ and all the elements in ***R***_sp_ and ***R***_ss_ are real, only the real part of the transfer functions (***Z***_sp_ and ***Z***_ss_) are useful in the simulations and they are calculated by using the theoretical modal superposition method^30^. The real part of transfer functions converges with the number of modal terms included and a total of 60 modal terms are used in the simulations. Equation (2) is used to obtain the optimized strengths of secondary loudspeakers, then the sound reduction can be obtained, which is defined as the difference between sound power level without and with the virtual sound barrier system. *β* is set as 1.0 to constrain the control effort in the simulations.

### Data availability

All data generated or analyzed during this study are included in this published article (and its Supplementary Information files).

## Electronic supplementary material

Supplementary information
