# Supplementary material for: Controlling sound radiation through an opening with secondary loudspeakers along its boundaries
Source: Sci Rep. 2017 Oct 17;7:13385. doi: 10.1038/s41598-017-13546-2 (PMC5645455; doi:10.1038/s41598-017-13546-2)
Supplement: Supplementary file 1 — Supplementary information [file 41598_2017_13546_MOESM1_ESM.pdf]

Supplementary information for

“Controlling sound radiation through an opening with secondary loudspeakers along its boundaries”

Shuping Wang[1], Jiancheng Tao[1,]*, Xiaojun Qiu[2]

*Correspondence to: jctao@nju.edu.cn

Supplementary Discussion

Planar active sound barrier

According to the Huygens' Principle, every point at a wave front may be considered as the source of secondary wavelets with a speed equal to the speed of the waves. This is the theoretical basis of planar active sound barriers. In a planar active sound barrier system, the loudspeakers are distributed over the entire opening as shown in Supplementary Fig. S1a, and they can be regarded as the sources of secondary wavelets, thus sound radiation through the opening can be reduced as long as there are sufficient of them. In the simulations, 32 loudspeakers are evenly distributed in *x*-*y* plane at the height of 0.448 m, as shown in Supplementary Fig. S1b, and the picture of the experimental setup is in Supplementary Fig. S1c.

Number of modal terms used in the simulations

In the simulations, we used the sound power of the system as the cost function, which can be calculated by[S1]

$$J = \frac{1}{2}\left\{\mathrm{Re}[q_{\mathrm{p}}^{\mathrm{H}} p_{\mathrm{p}}] + \mathrm{Re}[\boldsymbol{q}_{\mathrm{s}}^{\mathrm{H}} \boldsymbol{p}_{\mathrm{s}}]\right\} + \beta \boldsymbol{q}_{\mathrm{s}}^{\mathrm{H}} \boldsymbol{q}_{\mathrm{s}}\,, \tag{S-1}$$

where Re[] means the real part of the value in the square brackets. p_{p} and $\boldsymbol{p}_{\mathrm{s}}$ are the sound pressure at the positions of point sources. By minimizing Eq. (S-1), the optimized strengths of the loudspeakers can be obtained[S1]

$$\boldsymbol{q}_{\mathrm{s}} = -(\boldsymbol{R}_{\mathrm{ss}} + \beta \mathbf{I})^{-1} \boldsymbol{R}_{\mathrm{sp}} q_{\mathrm{p}}\,. \tag{S-2}$$

In Eq. (S-2), $\boldsymbol{R}_{\mathrm{ss}}$ is the real part of the acoustic transfer function matrix between the secondary loudspeakers, R_{sp} is the real part of the acoustic transfer function vector from

the primary sound source to the secondary loudspeakers, and q_p is the strength of the primary sound source. **I** is an identity matrix and β is a positive real number to constrain the strengths of secondary loudspeakers. In the simulations, we set q_p as 10^{-4} $\mathrm{m^3/s}$, so $\boldsymbol{q}_\mathrm{s}$ is a real vector.

Since q_p is a real number and all the elements in $\boldsymbol{q}_\mathrm{s}$ are real, whether the sound power of the system (Eq. (S-1)) can be accurately calculated only depends on the real part of p_p and $\boldsymbol{p}_\mathrm{s}$. The modal superposition method is applied to obtain the sound pressure. The sound pressure at $\boldsymbol{r}(x, y, z)$ inside the open cavity is calculated by[S2]

$$p_{\mathrm{in}}(\boldsymbol{r},\omega)=\sum_{n=1}^{N}(P_n^{\mathrm{i}}\mathrm{e}^{-\mathrm{j}k_{nz}z}+P_n^{\mathrm{r}}\mathrm{e}^{\mathrm{j}k_{nz}z})\phi_n(x,y)+\iiint_{\mathrm{V}}\mathrm{j}\rho_0\omega q(\boldsymbol{r}_\mathrm{s})G_\mathrm{A}dV\,, \tag{S-3}$$

in which

$$\phi_n(x,y)=\cos\frac{n_x\pi}{l_x}x\cos\frac{n_y\pi}{l_y}y\,, \tag{S-4}$$

$$G_\mathrm{A}=\frac{-\mathrm{j}}{2S}\sum_{n=1}^{N}\frac{\phi_n(x,y)\phi_n(x_0,y_0)}{\Lambda_n k_{nz}}\exp(-\mathrm{j}k_{nz}\left|z-z_0\right|)\,, \tag{S-5}$$

N is the number of modal terms that we used for the calculation of sound pressure.

In the near field of a point source, the sound pressure becomes intense, but we only focus on the real part of sound pressure, which converges with N. Supplementary Figure S2 shows the real and imaginary part of the sound pressure generated by a point source at its position. Supplementary Figures S2a and S2b are the real and imaginary part of the sound pressure at (0.1, 0.1, 0.1) m (the primary sound source) and Supplementary Figs. S2c and S2d are the results at (0.03, 0.03, 0.548) m (one of the secondary sources). The frequency of interest is 1000 Hz. It is clear that while the imaginary part of the sound

pressure diverges as the modal terms increases, the real part converges. It can be seen from Supplementary Figs. S2a and S2c that about 30 modal terms are sufficient to accurately calculate the real part of the sound pressure. We included 60 terms in our simulations (the error between 150 modal terms are only 0.38 % and 0.57 %), so the simulation results are valid.

The advantages of a double layered loudspeaker system

We employed a double layered loudspeaker system because it performs much better than a single layer system with the same number of secondary loudspeakers. We compared a double layered and a single layer system by simulations and experiments. Supplementary Figure S3 shows the positions of single layer loudspeakers in *x*-*y* plane and the experimental setup. The 32 loudspeakers are at the height of 0.448 m in the single layer system. In the double layer system, 32 loudspeakers are distributed at the same positions as in the manuscript. Both the simulated and experimental results of the sound power reduction achieved by the two systems are shown in Supplementary Fig. S4. It is clear that the sound power reduction the double layered loudspeaker system achieved is much higher than the single layer system, and that’s why we employed a double layered loudspeaker array instead of a single layer system.

Supplementary Figure S5 shows the sound power level without and with 48 secondary loudspeakers at the edge when they are divided into different numbers of layers. We can see that multi-layer loudspeaker arrays achieve much better sound reduction performance than a single layer; however, it is not that the more layers, the better.

Noise reduction with different layer heights

No matter which heights the 2 loudspeaker arrays are set at, the virtual sound barrier can achieve satisfactory sound power reduction over a wide frequency band. Supplementary Figures S6a and S6b show the sound power level without and with the virtual sound barrier under different configurations. Although the sound reduction is different when the loudspeakers are at different heights, all the sound power reductions are more than 35 dB.

More details about the experiments

The input port of the experimental system consists of 32 error microphones which are located over the entire opening. Their specific positions in *x*-*y* plane are shown in Supplementary Fig. S7a and Fig. S7b is a photo of the experimental setup of error microphones.

The virtual error sensor arrangement[S3-S4] will be investigated in the future to facilitate access through the opening. In the virtual error sensor arrangement, physical error microphones are implemented remote from the opening and the sound pressure at the opening is predicted by analytical or numerical methods and minimized to reduce the sound power radiation through the opening.

In the experiments, the expectation of the sum of the squared amplitude of the sine and cosine component of all the 32 error signals is defined as the cost function[S5-S6]

$$J = E\left[\sum_{\mathrm{m}=1}^{32}(A_{\mathrm{esm}}^{2}(n)+A_{\mathrm{ecm}}^{2}(n))\right]. \qquad \text{(S-6)}$$

The lth output of the active controller is

$$y_l(n) = A_{csl}(n)\sin(\omega n) + A_{ccl}(n)\cos(\omega n)\,, \tag{S-7}$$

where $l = 1, 2, \ldots, 32$, and $A_{csl}(n)$ and $A_{ccl}(n)$ are updated by

$$A_{csl}(n+1) = A_{csl}(n) - 2\mu\sum_{m=1}^{32}(A_{esm}(n)C_{sslm}(n) + A_{ecm}(n)C_{sclm}(n))\,, \tag{S-8a}$$

$$A_{ccl}(n+1) = A_{ccl}(n) - 2\mu\sum_{m=1}^{32}(A_{esm}(n)C_{cslm}(n) + A_{ecm}(n)C_{cclm}(n))\,, \tag{S-8b}$$

where C_{sslm}, C_{sclm}, C_{cslm} and C_{cclm} are the acoustic transfer functions between the mth error microphone to the lth loudspeaker, which can be obtained with the online cancellation path modelling method.

With an appropriate step size μ, Equation (S-8) converges to the optimized solution. In the experiments, 10 microphones are located on a hemisphere frame of 1.5 m radius according to ISO 3744 to measure the sound power level of the system, and the positions are listed in Supplementary Table S1[S7].

The sound power level (SWL) of the system with the virtual sound barrier working is calculated by the 10 sound pressure levels L_{pi} ($i = 1, 2, \ldots, 10$) at the 10 microphones and the area of the hemisphere S

$$\mathrm{SWL}_{\mathrm{with}} = \overline{L}_{\mathrm{p}} + 10\lg\left[\frac{S}{S_0}\right], \tag{S-9}$$

where $S_0 = 1\ \mathrm{m}^2$, and $\overline{L}_{\mathrm{p}}$ is the average sound pressure at the 10 points

$$\overline{L}_{\mathrm{p}} = 10\lg\left[\frac{1}{10}\sum_{i=1}^{N}10^{0.1L_{pi}}\right]. \tag{S-10}$$

The sound power reduction of the system is defined as the difference of the sound power level without and with the virtual sound barrier ($SWL_{\_without}$ and $SWL_{\_with}$)

$$\mathrm{NR} = \mathrm{SWL}_{\mathrm{without}} - \mathrm{SWL}_{\mathrm{with}}\,, \tag{S-11}$$

which is used to evaluate the performance of the system, and the sound power reduction is calculated with Eq. (S-11) in the manuscript and the supplementary information.

The active noise control algorithm applied in the experiments

Take a single channel system as an example. Since the primary sound source produces tonal sound field, the expectation of the sum of the squared amplitude of the sine and cosine component of the error signal is defined as the cost function[S5-S6]

$$J = E\left[A_{\mathrm{es}}^2(n) + A_{\mathrm{ec}}^2(n)\right], \tag{S-12}$$

where $A_{\mathrm{es}}(n)$ and $A_{\mathrm{ec}}(n)$ are calculated by

$$A_{\mathrm{es}}(n) = \frac{2}{N}\sum_{i=1}^{N}\sin(\omega(n-N+\mathrm{i}))e(n-N+i)\,, \tag{S-13a}$$

$$A_{\mathrm{ec}}(n) = \frac{2}{N}\sum_{i=1}^{N}\cos(\omega(n-N+\mathrm{i}))e(n-N+i)\,. \tag{S-13b}$$

After applying the gradient method to minimize the cost function in Eq. (S-12), the output of the active controller can be obtained

$$y(n) = A_{\mathrm{cs}}(n)\sin(\omega n) + A_{\mathrm{cc}}(n)\cos(\omega n)\,, \tag{S-14}$$

where $A_{\mathrm{cs}}(n)$ and $A_{\mathrm{cc}}(n)$ are updated by

$$A_{\mathrm{cs}}(n+1) = A_{\mathrm{cs}}(n) - 2\mu(A_{\mathrm{es}}(n)C_{\mathrm{ss}}(n) + A_{\mathrm{ec}}(n)C_{\mathrm{sc}}(n))\,, \tag{S-15a}$$

$$A_{cc}(n+1) = A_{cc}(n) - 2\mu(A_{es}(n)C_{cs}(n) + A_{ec}(n)C_{cc}(n)) . \qquad \text{(S-15b)}$$

μ is the step size of the update. $C_{ss}(n)$, $C_{sc}(n)$, $C_{cs}(n)$ and $C_{cc}(n)$ are the cancellation path transfer functions which can be calculated with on-line cancellation path modelling method. With an appropriate μ, Equation (S-15) converges to the optimized solution.

The internally synthesized tonal signal is synthesized as the reference signal, so we don't need a reference microphone. Advantages of the algorithms also include fast convergence speed and low computation load.

Noise reduction for general noise sources

We presented the results under the simplest condition that the primary source is a point source because the loudspeaker can be regarded as a point source within relatively low frequency range and this enables us to quantify and compare the simulation and experiment results. The system is also effective when the primary source is more complicated. Supplementary Figure S8 shows the sound power level without and with the virtual sound barrier when the primary source is a dipole, longitudinal quadrupole, lateral quadrupole, a line source and plane source, respectively.

Two point sources at (0.1, 0.1, 0.1) m and (0.1, 0.13, 0.1) m with opposite strengths (10^{-4} m^3/s and -10^{-4} m^3/s) are applied to simulate a dipole. Point sources at (0.1, 0.1, 0.1) m and (0.1, 0.16, 0.1) m with the strength 0.1 m^3/s and a third point source at (0.1, 0.13, 0.1) m with the strength -0.2 m^3/s are applied to simulate the longitudinal quadrupole. In the lateral quadrupole, the strengths of the two point sources at (0.1, 0.1, 0.1) m and (0.13, 0.13, 0.1) m are 0.1 m^3/s, and the strengths of the other two point sources at (0.1, 0.13, 0.1) m and (0.13, 0.1, 0.1) m are $-0.1 m^3/s$.

42 point sources of the same strength 10^{-4} m^3/s are located in a line from (0.01, 0.10, 0.10) m to (0.42, 0.10, 0.10) m with an interval of 0.01 m and to simulate a line source. 64 point sources of the same strength 10^{-4} m^3/s located in a 0.07 m × 0.07 m square with an interval of 0.01 m to simulate the surface source.

The virtual sound barrier system still has 32 secondary sources at the edge of two layers as mentioned in the manuscript. It is clear in Supplementary Fig. S8 that the sound power reduction below 1000 Hz in all the cases are more than 30 dB.

We did 3 experiments on more complicated situations when there are multiple primary sources emitting sound energy at different frequencies:

(1) A line primary source with 6 loudspeakers, as shown in Supplementary Fig. S9a. These loudspeakers radiate sound energy at the same frequency. We still apply the waveform synthesis algorithm and tonal sound of the same frequency is used as the reference signal by the active controller[S5-S6]. The sound power level without and with the virtual sound barrier and the sound power reduction are shown in Supplementary Table S2.

(2) Two point sources at different locations simultaneously emitting acoustic energy at different frequencies. These two point sources are at (0.1, 0.1, 0.1) m and (0.2, 0.2, 0.2) m and the exciting frequency is f_1 and f_2, respectively. The photo of the experimental setup is shown in Supplementary Fig. S9b. The internally synthesized tonal signals at f_1 and f_2 are used as the reference signal[S5-S6]. The sound power level at these two frequencies with and without the virtual sound barrier and the sound power reductions are shown in Supplementary Table S3.

(3) Three point sources at different locations emitting acoustic energy at 3 different frequencies 600 Hz, 700 Hz and 800. The experimental setup is shown in Supplementary Fig. S9c. The sound power level at the 3 frequencies with and without the virtual sound barrier and the sound power reductions are shown in Supplementary Table S4.

We can see that all these sound power reductions with multiple sound sources in Supplementary Tables S2, S3 and S4 are more than 10 dB, but less than that with a single primary source. The reason is that dealing with 3 frequencies simultaneously puts very heavy computation load on the active controller used in the experiments, which deteriorates the performance of the system. Due to the constraints of the hardware and algorithm used in the experiments, 10-20 dB is almost the best result we could achieve with the current system. Although the noise reduction is not as good as that with a single primary source, 10 dB noise reduction is sufficient for demonstrating the feasibility of the concept proposed in the paper. These experiments demonstrate the feasibility of the virtual sound barrier system when the primary noise source is a more general one.

We did some further numerical simulations on the experimental setup with 6 primary sources as shown in supplementary Fig. S9a. Supplementary Figure S10a shows the sound power reduction in numerical simulations when all the 6 primary sources (P_1, P_2, …, P_6 as shown in supplementary Fig. S10b) with the same strengths emit tonal sound simultaneously and when only one of them radiates sound. Supplementary Figure S10a shows clearly that the sound power reductions are almost the same when there are 1 or 6 primary sources. This demonstrates that the system works effectively in broad frequency range for tonal sound no matter how complicated the primary sound field is.

The double layered secondary loudspeaker system can achieve effective control of low frequency sound in experiments for complicated primary sound fields, but the experimental results at high frequencies is not as good as that in low frequency range due to the constraints of hardware at present. We believe its performance will be improved if more powerful active controller can be obtained.

Alternative error sensor strategies

The error microphones are distributed over the entire opening in the experiments in the paper, but there are other alternative error sensor arrangements to avoid putting them in the pathway of the opening. As long as double-layered secondary sources at the edge of the opening work effectively, the system has the possibility to be invisible. Supplementary Figure S11a shows the sound power reduction when 32 error microphones are in the far field on a semi-sphere with a radius of 5 m of which the center is at the center of the opening. We can see that error microphones in the far field also work effectively.

Supplementary Figure S11b shows the sound power reduction when the 32 error microphones are at the edge of two layers, 0.568 m and 0.588 m planes. The sound power reduction is compared with that when error microphones are at the edge of a single layer 0.588 m and over the entire plane of 0.588 m. It is clear that just like double-layered secondary sources can improve the noise reduction performance, using double-layered error microphones achieves higher sound power reduction than using single-layered error microphones at the edge. Therefore, using double-layered error microphones is also

feasible to achieve effective global control, especially at low frequency range, when active noise control works more effectively.

Supplementary Figures

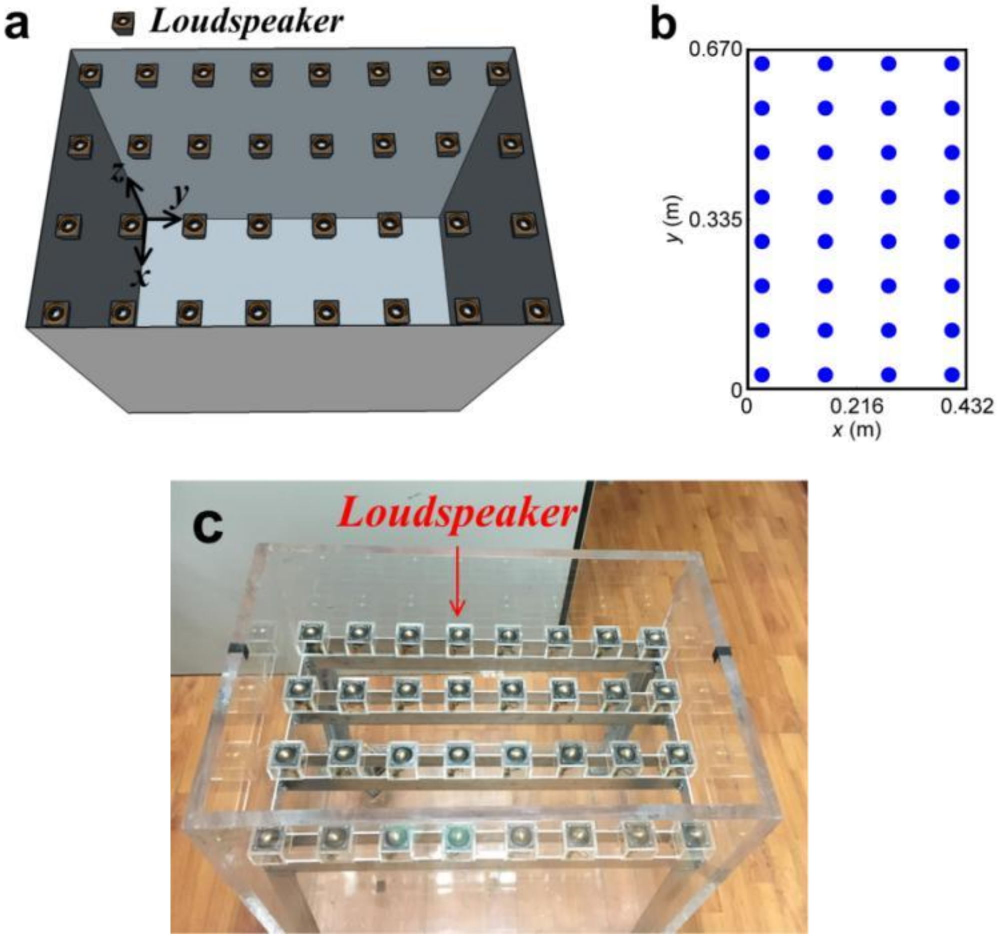

Supplementary Figure S1 | (**a**) Schematic diagram of the secondary loudspeakers in a planar active sound barrier system. (**b**) Positions of the 32 loudspeakers in the planar active sound barrier in simulations. (**c**) Experimental setup of the loudspeakers in the planar active sound barrier system.

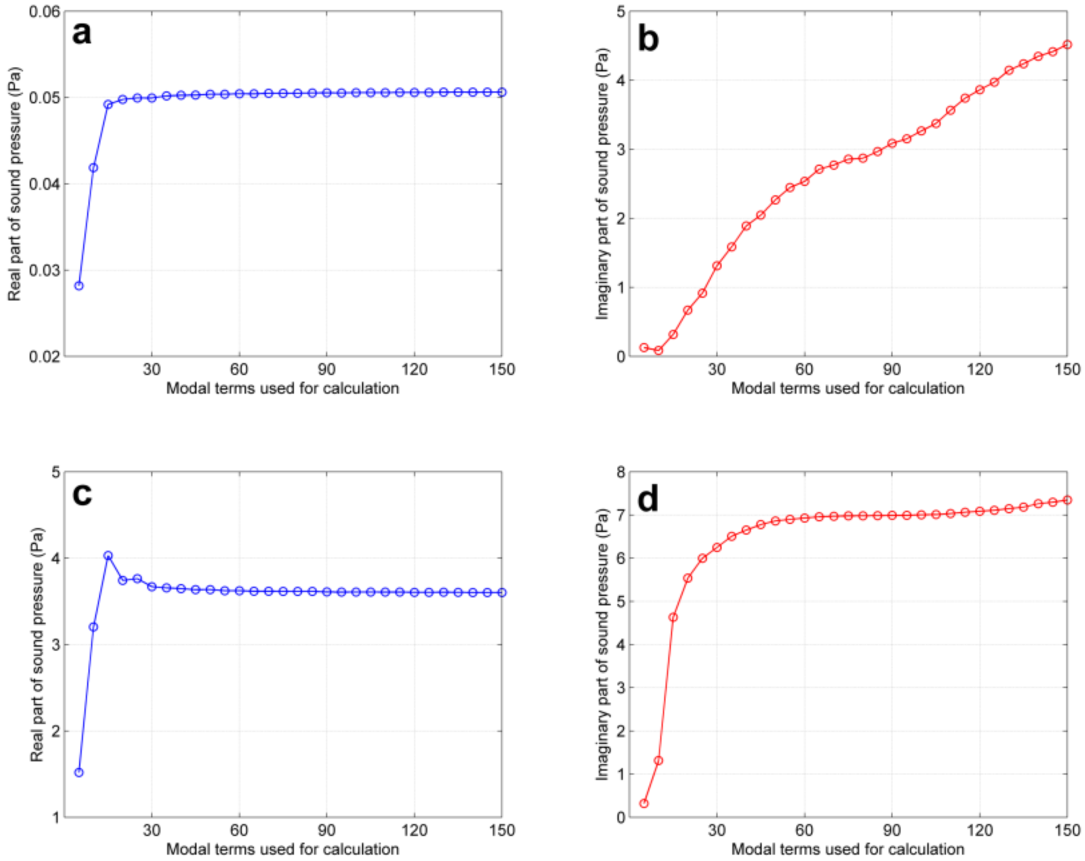

Supplementary Figure S2 | The sound pressure at the position of the sound source. (**a**) Real part, the sound source is at (0.1, 0.1, 0.1) m. (**b**) Imaginary part, the sound source is at (0.1, 0.1, 0.1) m. (**c**) Real part, the sound source is at (0.03, 0.03, 0.548) m. (**d**) Imaginary part, the sound source is at (0.03, 0.03, 0.548) m.

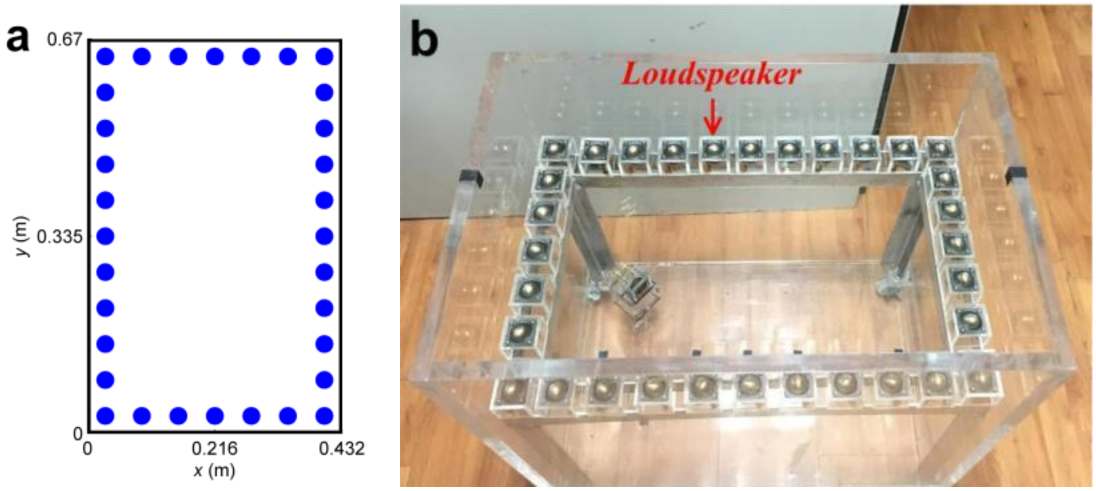

Supplementary Figure S3 | (a) Positions of loudspeakers in a single layer system. **(b)** Picture of the experimental setup of single layer loudspeakers.

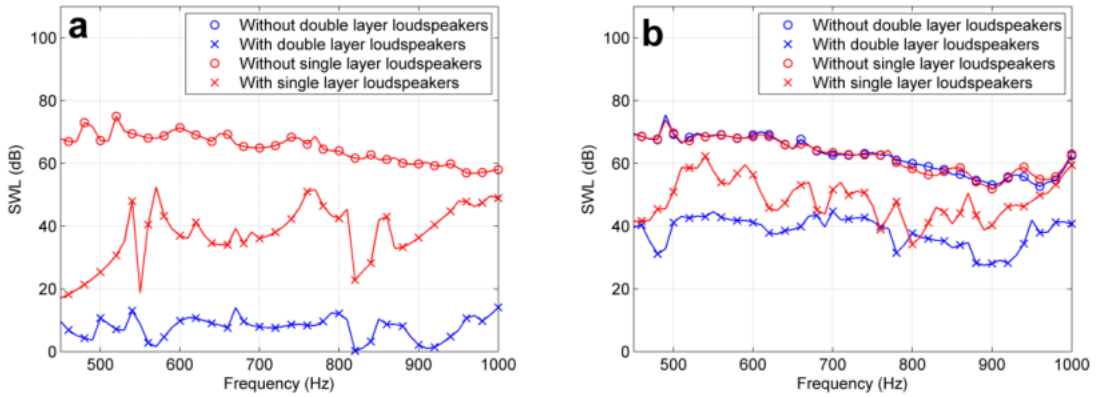

Supplementary Figure S4 | The sound power level (SWL) without and with the double layered loudspeaker array and single layered array. (**a**) Simulation results. (**b**) Experimental results.

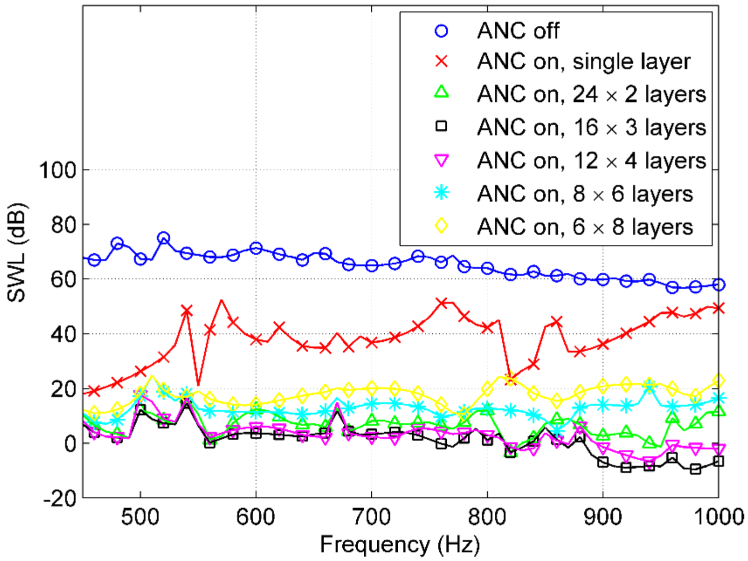

Supplementary Figure S5 | The sound power level (SWL) when active noise control (ANC) is off and on with different numbers of loudspeaker layers.

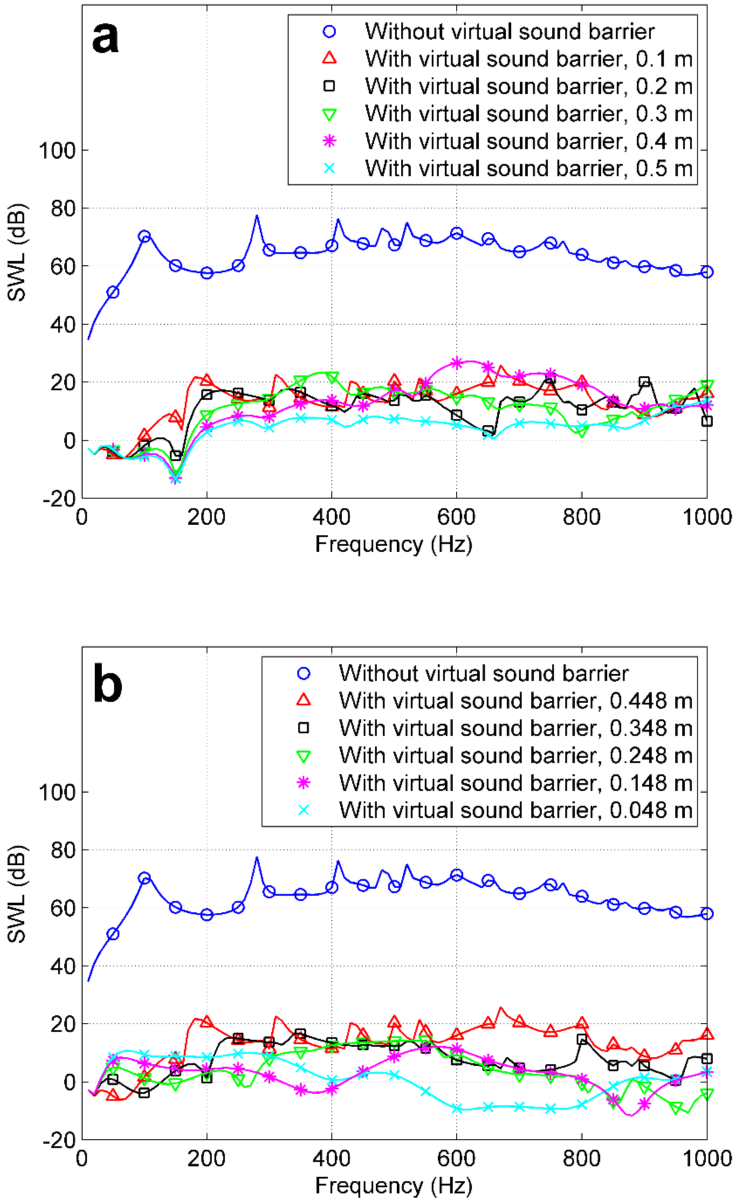

Supplementary Figure S6 | The sound power level without and with the virtual sound barrier. (**a**) One layer of loudspeakers is at the height of 0.548 m, and the distance between the two layers is 0.1 m, 0.2 m, 0.3 m, 0.4 m and 0.5 m. (**b**) The distance between two layers is fixed as 0.1 m, and the lower layer is at the height of 0.448 m, 0.348 m, 0.248 m, 0.148 m and 0.048 m.

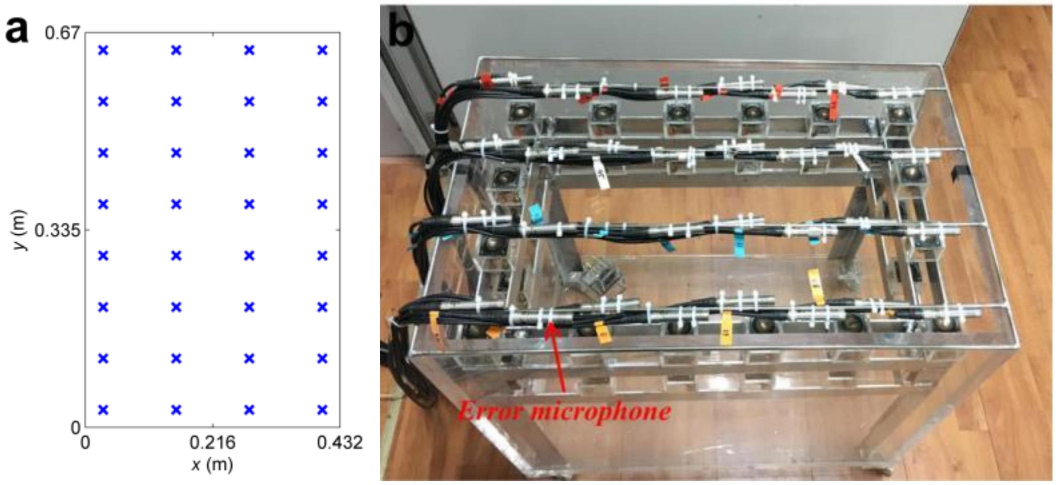

Supplementary Figure S7 | (a) Schematic diagram of the positions of 32 error microphones. (**b**) Photo of the experimental setup of error microphones.

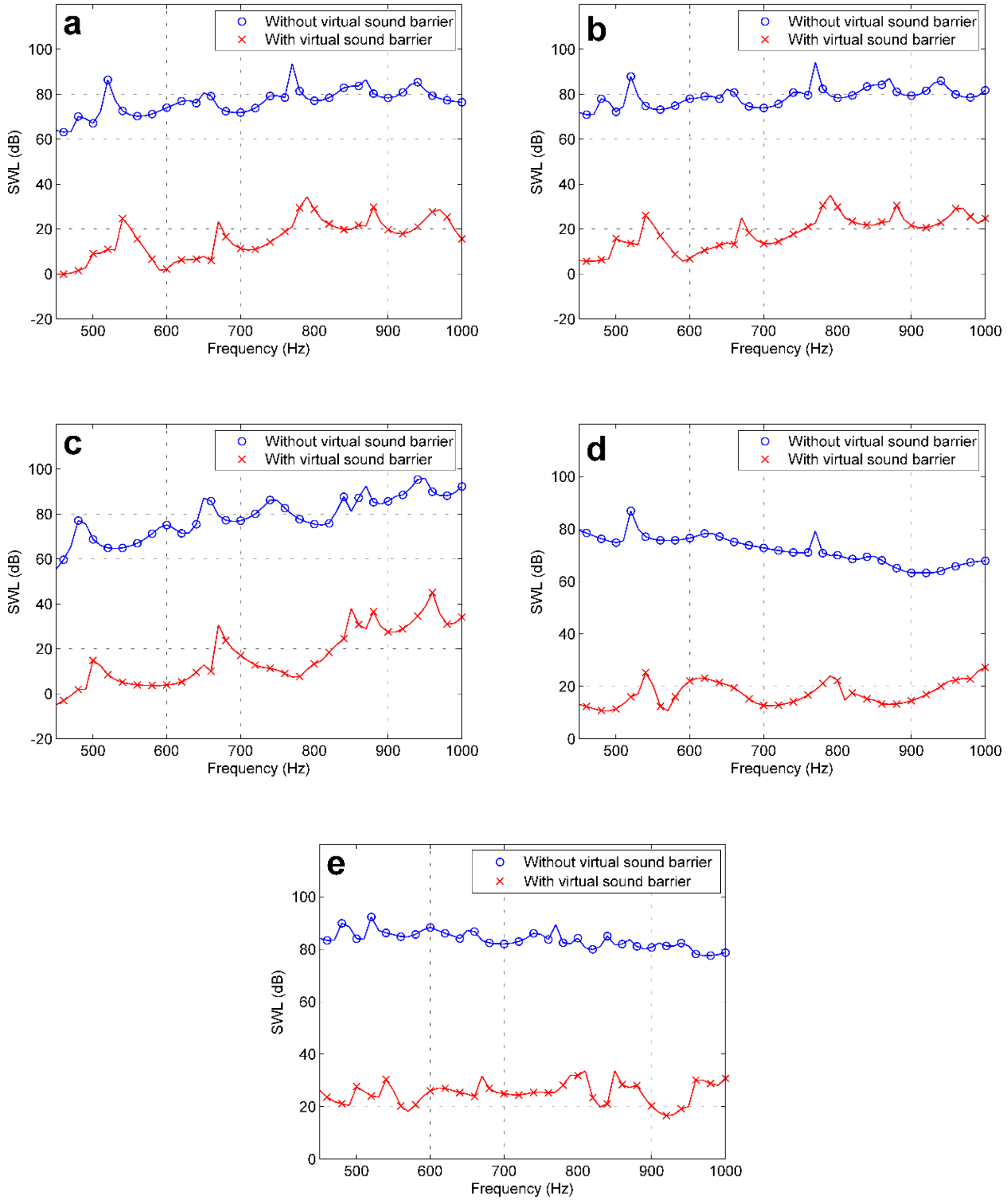

Supplementary Figure S8 | The sound power level (SWL) without and with the virtual sound barrier. (**a**) Dipole. (**b**) Longitudinal quadrupole. (**c**) Lateral quadrupole. (**d**) Line source. (**e**) Plane source.

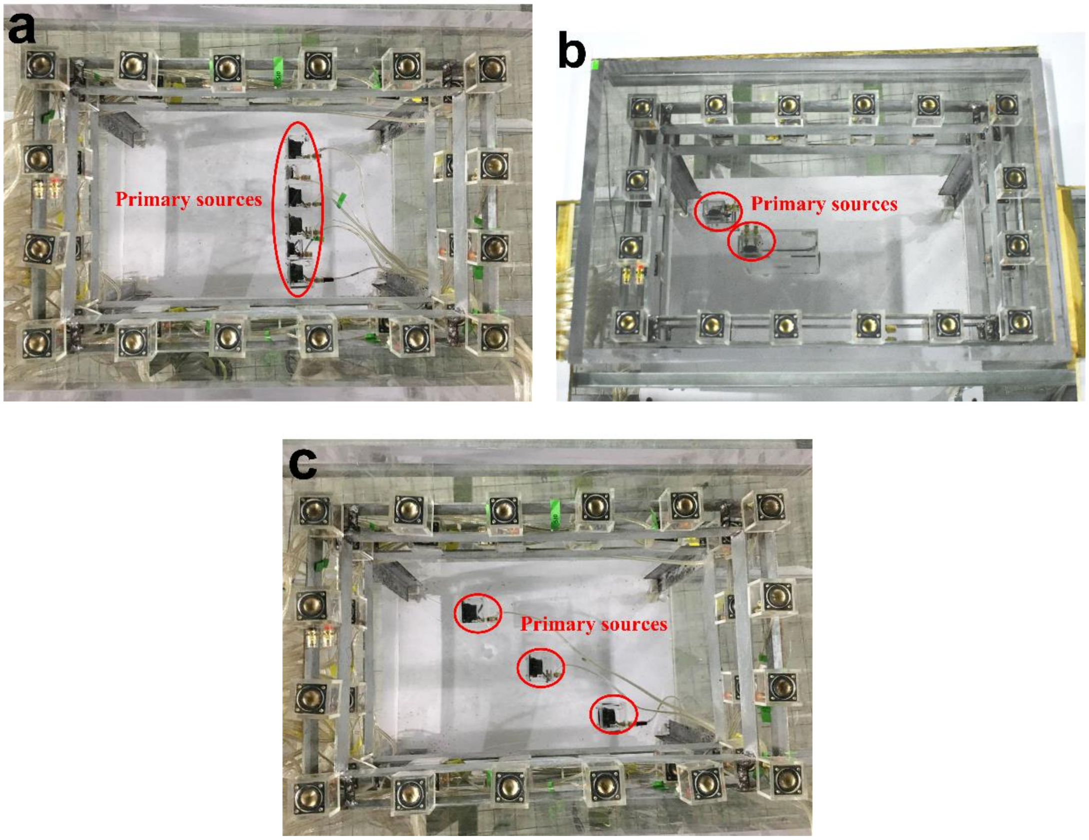

Supplementary Figure S9 | Pictures of the experimental setup. (**a**) A line primary sources with 6 loudspeakers, which emit sound energy at the same frequency. (**b**) Two primary sources, which emit sound energy at frequencies f_1 and f_2. (**c**) Three primary sources, which emit sound energy at 600 Hz, 700 Hz and 800 Hz, respectively.

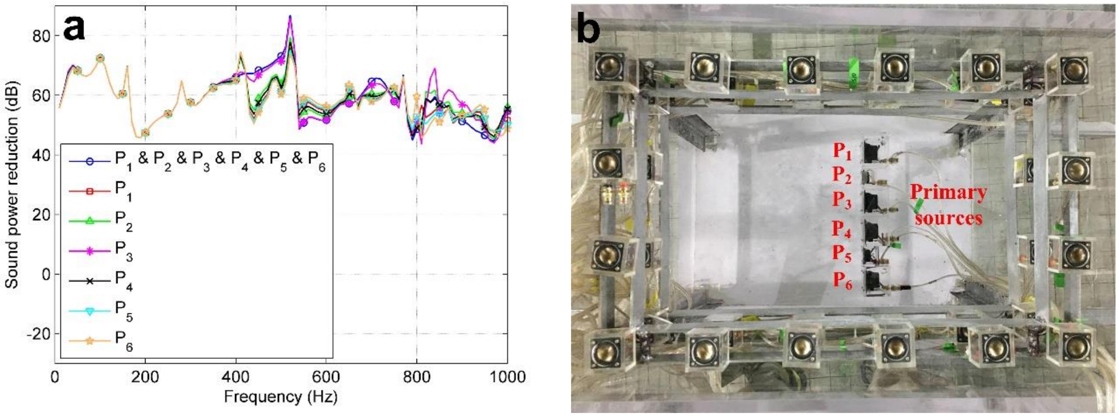

Supplementary Figure S10 | (**a**) The sound power reduction when all of the 6 primary sources work simultaneously or only one of them radiates sound. (**b**) The positions of the 6 primary sources.

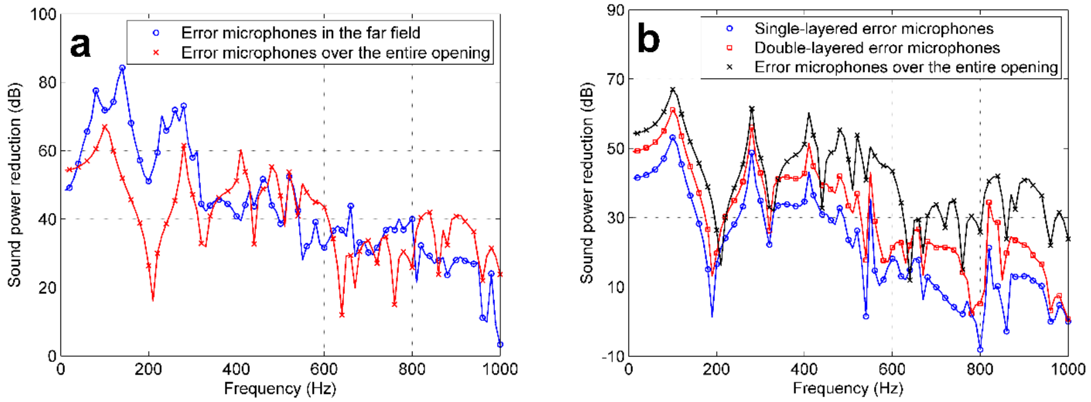

Supplementary Figure S11 | (**a**) The sound power reduction when 32 error microphones are implemented in the far field and over the entire opening. (**b**) The sound power reduction when 32 error microphones are implemented at the edge of a single layer, two layers and over the entire opening.

Supplementary Table S1 Microphone positions for free field over a reflecting surface.

No.	*x*/*r*	*y*/*r*	*z*/*r*
1	–0.99	0	0.15
2	0.50	–0.86	0.15
3	0.50	0.86	0.15
4	–0.45	0.77	0.45
5	–0.45	–0.77	0.45
6	0.89	0	0.45
7	–0.33	0.57	0.75
8	–0.66	0	0.75
9	0.33	0	0.75
10	0	–0.57	1.00

Supplementary Table S2 The sound power level without and with the virtual sound barrier and the sound power reduction when there is a line primary source with 6 loudspeakers emitting sound energy at the same frequency.

Frequency (Hz)	Sound power level (dB)		Sound power reduction (dB)
	Without virtual sound barrier	With virtual sound barrier	
500	71.9	45.8	26.1
650	76.8	43.8	33.0
800	69.3	53.8	15.5
900	70.5	54.6	15.9

Supplementary Table S3 The sound power level without and with the virtual sound barrier and the sound power reduction when there are two primary sources emitting sound energy at different frequencies.

Frequency (Hz)	Sound power level (dB)				Sound power reduction (dB)	
	Without virtual sound barrier		With virtual sound barrier			
	f_1	f_2	f_1	f_2	f_1	f_2
f_1 = 450, f_2 = 900	70.0	66.2	42.1	51.0	27.9	15.2
f_1 = 500, f_2 = 1000	68.7	70.0	45.0	49.0	23.7	21.0
f_1 = 500, f_2 = 600	68.6	61.8	45.4	46.0	23.2	15.8
f_1 = 500, f_2 = 700	68.7	66.6	46.0	42.0	22.7	24.6

Supplementary Table S4 The sound power level without and with the virtual sound barrier and the sound power reduction when there are 3 primary sources emitting sound energy at 600 Hz, 700 Hz and 800 Hz, respectively.

3requency (Hz)	Sound power level (dB)		Sound power reduction (dB)
	Without virtual sound barrier	With virtual sound barrier	
600	70.4	47.3	23.1
700	68.1	54.8	13.3
800	72.0	60.2	11.8

References

S1. Elliott, S., Joseph, P. Nelson, P. & Johnson, M. Power output minimization and power absorption. *J. Acoust. Soc. Am.* **90**, 2501-2512 (1991).

S2. Wang, S., Tao, J. & Qiu, X. Performance of a planar virtual sound barrier at the baffled opening of a rectangular cavity. *J. Acoust. Soc. Am.* **138**, 2836-2847 (2015).

S3. Garcia, J., Elliott, S. & Boucher, C. Generation of zones of quiet using a virtual microphone arrangement. *J. Acoust. Soc. Am.* **101**, 3498-3516 (1997).

S4. Rafaely, B., Elliott, S. & Garcia, J. Broadband performance of an active headrest. *J. Acoust. Soc. Am.* **102**, 787-793 (1999).

S5. Qiu, X. & Hansen, C. An algorithm for active control of transformer noise with on-line cancellation path modeling based on the perturbation method. *J. Sound. Vib.* 240, 647-665 (2001).

S6. Qiu, X., Li, X., Ai, Y. & Hansen, C. A waveform synthesis algorithm for active control of transformer noise: implenmentation. *Appl. Acoust.* 63, 467-479 (2002).

S7. ISO 3744: Acoustics-Determination of sound power levels of noise sources using sound pressure - Engineering method in an essentially free field over a reflecting plane. (1994).
